# Using the Past to Maximize the Success Probability of Future Anti-Viral Vaccines

**DOI:** 10.3390/vaccines8040566

**Published:** 2020-10-01

**Authors:** Philip Serwer

**Affiliations:** Department of Biochemistry and Structural Biology, The University of Texas Health Center, San Antonio, TX 78229-3900, USA; serwer@uthscsa.edu; Tel.: +1-210-567-3765

**Keywords:** immune system complexity, influenza, pandemics, virus-caused, SARS-CoV-2, vaccine development strategy

## Abstract

Rapid obtaining of safe, effective, anti-viral vaccines has recently risen to the top of the international agenda. To maximize the success probability of future anti-viral vaccines, the anti-viral vaccines successful in the past are summarized here by virus type and vaccine type. The primary focus is on viruses with both single-stranded RNA genomes and a membrane envelope, given the pandemic past of influenza viruses and coronaviruses. The following conclusion is reached, assuming that success of future strategies is positively correlated with strategies successful in the past. The primary strategy, especially for emerging pandemic viruses, should be development of vaccine antigens that are live-attenuated viruses; the secondary strategy should be development of vaccine antigens that are inactivated virus particles. Support for this conclusion comes from the complexity of immune systems. These conclusions imply the need for a revision in current strategic planning.

## 1. Introduction

Pandemic-causing viruses have become a major problem for all people. Influenza viruses and coronaviruses are the viral purveyors of our most destructive, recent pandemics. Both have a single-stranded RNA (ssRNA) genome and a membrane envelope. Vaccination is the most comprehensive clinical response for ssRNA, membrane-enveloped viruses and others [1,2,3]. 

To help optimize rapid vaccine response to emerging virus-caused pandemics, I outline here successful vaccine-producing strategies used in the past for ssRNA, membrane-enveloped viruses. I separately also outline successful vaccine-producing strategies used in the past for other viruses. Successful, sustained-use vaccines of the following types have been developed: live attenuated (LA), inactivated whole virus (IW), inactivated/split into subunits (IS) and, more recently, recombinant subunit (RS) and live recombinant (LR). 

## 2. Vaccines Against Diseases Caused by ssRNA, Membrane-Enveloped Viruses 

### 2.1. Past Successes

I initially focus on diseases caused by ssRNA, membrane-enveloped viruses. Comprehensively for humans and excluding animals, the diseases are the following (vaccine type, in order of development, followed by the approximate year(s) of the first vaccine introduction): rabies (LA, IW; 1885 [4,5,6]), yellow fever (LA; 1935-1937 [4,7,8]), Japanese B encephalitis (IW, LA; 1944 [4,9,10]), influenza (LA, IW, IS, RS; 1945 [4,11,12,13]), measles (LA; 1962 [4,14,15]), mumps (LA; 1966 [4,16,17]) and German measles, or rubella (LA; 1969-1979 [4,18,19]) (Table 1). Vaccine for Zaire ebolavirus [20] appears to be next (LR; 2020 [21]). The latter vaccine has an ebolavirus membrane protein expressed in the membrane of vesicular stomatitis virus [21]. 

The following points are noted: (1) The LA, IW and LR vaccines all have in common the use of an antigen that is relatively “native”, i.e., that is in a context that resembles the context in the virus that causes the disease. (2) A LA vaccine is successful for all of the above. (3) The time interval between the most recent vaccine on this list and its successor is, at a minimum, 41 years. This interval coincides, approximately, with the time interval in which modern molecular biology-based techniques become available for vaccine development. For example, the first vaccine produced by recombinant DNA technology was introduced in 1986, as discussed in Section 3 below.

Although molecular biology-based techniques have had some successes, these successes were achieved only in the context of at least partially successful work on more traditional techniques. Work on influenza virus vaccines is an example, above. Other examples are presented below.

### 2.2. Limitations Encountered 

Although LA vaccines are the most native, they have the obvious risk of insufficient attenuation. The large contingent of LA vaccines (Table 1 and Table 2) suggests that sufficient attenuation, without losing antigenicity, is often possible without understanding the structural details involved. Improvements should be possible by determining viral structures and using these structures to direct attenuating mutations. However, departure from a LA vaccine also has risks and these appear to be more difficult to evaluate, given the examples presented below. The first example is an approved, quadrivalent, LR (also called chimeric) vaccine for dengue, caused by a ssRNA, membrane-enveloped virus. Each vaccine strain, corresponding to the four pathogenic strains, has a yellow fever virus genome, with attenuated genes for two dengue virus membrane proteins inserted in place of yellow fever counterparts [22,23]. One result is that the vaccine aggravates disease for some natural infection-naïve human recipients, including children [24,25]. A possible reason is vaccine stimulation of non- or weakly-neutralizing antibodies that promote uptake of virus into macrophages [26]. This phenomenon is often called antibody dependent (disease) enhancement (ADE).

Similarly, an IW vaccine for respiratory syncytial virus converted a mild (upper respiratory) infection into a serious, sometimes fatal, one in humans [27,28,29]. Antibodies were induced, but they were primarily non-neutralizing [28,29]. Respiratory syncytial virus is a ssRNA, membrane-enveloped virus [28,29]. LA vaccines for respiratory syncytial virus did not have this effect. However, LA vaccines do not yet have a usable combination of immunogenicity and attenuation [29].

IW vaccine aggravation of disease has also been observed when successful LA vaccines have been obtained for ssRNA, membrane-enveloped viruses. IW vaccine for measles virus is an example [14]. This result is similar to the result with the dengue vaccine, except that no protective effects are observed. Projected reasons are not given. Finally, attempts to produce an IW vaccine for the rubella virus failed [19].

## 3. Vaccines Against Diseases Caused by Viruses of Other Types

Vaccines against viruses of other types include, comprehensively for humans, (1) LA vaccine against smallpox, caused by variola virus, a double-stranded (ds) DNA, membrane-enveloped virus (1796 [30,31]); (2) IW and LA and vaccines against poliovirus, a ssRNA, non-enveloped virus (1955 [4,32]); (3) IW vaccine against adenovirus, a double-stranded (ds) DNA, non-enveloped, virus (1956–1957 [4,33]); (4) LA vaccine against Marek’s disease of chickens, a cancer caused by a herpes-like dsDNA, membrane-enveloped virus (1971 [4,34]); (5) novel vaccines against hepatitis B virus, a dsDNA/ssDNA, membrane-enveloped virus (1981 [4,35,36]); (6) IW and LA vaccines against hepatitis A virus, a ssRNA, non-enveloped virus (1994 [4,37]); (7) LA vaccine against chickenpox (and shingles), caused by another herpes virus, varicella-zoster (1995 [4,38]); (8) LA vaccine against rotavirus, a segmented, dsRNA, non-enveloped virus (2006 [39,40]); and (9) an assembled, capsid-like, protein nanoparticle vaccine against several cancers caused by papilloma viruses, which are dsDNA, non-enveloped viruses (2006 [41,42]) (Table 2). The novel vaccines against hepatitis B virus started with a vaccine against assembled, blood-purified viral surface antigen ([4,35,43,44]). This vaccine was replaced, in 1986, by a recombinant protein vaccine that produced this antigen after expression of assembled capsid protein in yeast cells [4,43,44].

The dominance of LA vaccines is less for the viruses of Table 2. IW vaccines have a more prominent role, with adenovirus having only an IW vaccine. In addition, the more recent vaccine for hepatitis B virus is the first vaccine for which the antigen is obtained as a recombinant protein. I note that, although negative results are sometimes hard to find, a report does exist of failure of the recombinant hepatitis B vaccine when the antigen is expressed in bacterial, not yeast, cells [43]. 

## 4. Glycan Antigens: Vaccines for Bacterial Pathogens

### History: Anti-Bacterial Vaccines

Recent anti-bacterial vaccines generate a new perspective on anti-viral vaccines. The antigens of the initial anti-bacterial vaccines were attenuated or inactivated cells. Subsequently, inactivated bacterial toxins (toxoids) were used as antigens. Toxoids were originally made by treatment of toxins with formaldehyde (reviews [45,46]). Without going into detail about the early anti-bacterial vaccines, I summarize recent anti-bacterial vaccines made by conjugating bacterial polysaccharides to protein toxoids; the toxoids are adjuvant capable, especially for children [45,46] (tetanus toxoid and others are used [46]). More recently [46], conjugation to adjuvant-capable polysaccharides has been used. 

The toxoid-conjugated vaccines are against the following: *Haemophilus*-caused diseases [47], pneumococcal pneumonia, with successive vaccines active against increasing numbers of bacterial strains [48] and meningitis [49]. Apparently, the outer polysaccharide layers of the bacteria involved are reproducible, distinct, conformationally native and strain-specific enough for use in vaccines. Thus, if antigenic glycans are used in anti-viral vaccines, past experience suggests (but does not prove) that they will be less constrained than proteins to be in a native context.

In contrast, unassembled, protein-containing, subunit antigens have less frequently been used in successful anti-microbe vaccines, unless the vaccine was for a bacterial toxin, such as diphtheria toxin [45,46]. Protein antigens in anti-virus vaccines include the most recent hepatitis B vaccine antigen and some (but not most) influenza vaccine antigens (above). These appear, at first glance, to be exceptions to the native context preference rule for vaccine antigens.

However, if these protein-containing antigens either are assembled into virus capsid-like particles or are glycoproteins with glycan epitopes, then they can be interpreted as exceptions that support the rule. In the case of glycan epitopes, the reason is that protein-associated glycans (1) are hydrophilic [50,51] and project into the external solution, while they are on the surface of the S-layer of bacterial cells [51] and the surface of viruses [52], and (2) presumably do not depend for conformation on a lipid bilayer membrane. Thus, after purification, glycans, unlike membrane proteins, can be in the same (native) state as they are when microbe associated.

That the epitope for hepatitis B vaccine is more complex than unassembled, underivatized protein is implied by the following. The antigen of hepatitis B vaccines is known to be assembled to form a virus capsid-like particle; it is not the protein monomer [53,54]. Immunogenicity increases with the increasing level of glycan derivatization [55,56]. 

Similarly, the antigen for recombinant influenza vaccine is obtained from insect cells infected by a recombinant baculovirus [11,12,13]; the immunity generating importance of glycosylation is recognized [13]. In addition, the vaccine antigen for rabies is a glycoprotein [5,6]. Nonetheless, I find no evidence that either pure polysaccharide or polysaccharide-containing, conjugated vaccines have been attempted against viruses.

## 5. A Guideline Based on the Above: Support from Fundamental Considerations 

### 5.1. The Guideline 

In summary, the following guideline emerges from the above outline when extrapolated to new anti-virus vaccines, especially for ssRNA, enveloped viruses, including SARS-CoV-2. With the possible exception of a glycan antigen, maintaining the antigen in a native context is the optimal way to maximize the probability of a successful vaccine antigen. At this point, a native context implies one of the following vaccine types: LA (preferably), IW, LR. I have proposed a fourth, not-yet-tested way to maintain a native context by use of bacteriophages physically homologous to viral pathogens [57]. For the real world of today, the implied rhetorical question is the following. While we are throwing vaccine dice, why not follow an experience-of-the-past guideline to load the dice in our favor?

That apparently is not, in general, being done for SARS-CoV-2. Among the SARS-CoV-2 vaccines in phase 1 or phase 2 clinical trials in Europe and the United States, none are LA or IW in character. IW vaccines have reached phase 2 trials in China [58,59]. To the extent that these efforts in the United States and Western Europe are the products of strategy, one has to question the strategy.

### 5.2. Some Fundamentals

In addition, from my position as a specialist in phage assembly, I think that the above perspective is supported by the following fundamentals. Based on the literature, one fundamental aspect of the innate immune system is pattern recognition. The patterns discussed are usually the presence of viral molecules (uncapped mRNAs and dsRNAs, for example) that have a chemical composition different from that of host counterparts [60,61,62]. For example, Toll-like receptors in both the cytoplasmic membrane and endosomes have evolved to (1) recognize patterns of this type before the adaptive immune system starts to work and (2) initiate an adaptive immune response [63,64]. The topic of Toll-like receptors (at least 10 of them [63]) is complex. Toll-like receptors are not the only receptors involved [60,61,62].

Furthermore, immune system-recognized spatial patterns can be much larger than those known to be recognized by Toll-like receptors. For example, the mononuclear phagocyte system recognizes the size of opsonized foreign nanoparticles and removes > 40 nm particles to the liver and spleen [65,66]. 

In addition, the possibility exists that the temporal sequence of infection events is part of the pattern recognized, although I have not seen this possibility previously discussed. Temporal event sequencing is, for example, the mechanism for controlling the assembly of the complex tail of bacteriophage T4 [67,68]. Incompatibility with the temporal event sequencing program is a possible reason for suppression of immunity to other diseases generated by using measles vaccines at higher than optimal levels [14]. The hypothesis is that the innate immune system program includes recognizing progressively increasing amounts of virus, not the sudden presence of an amount larger than what one would naturally receive.

In view of this complexity, negative outcomes of using a relatively non-native vaccine are difficult to project and can include being blindsided by events that are missed in trials, as in the above cases of high-titer measles vaccines [14] and respiratory syncytial virus vaccines [28,29]. Finally, evidence exists that not all components of the innate immune system have been investigated as such. For example, evidence exists that amyloid-forming proteins are part of a mostly acellular innate immune system that causes neurodegenerative disease when malfunctioning [69].

The theoretical conclusion is that (1) the immune response has too much complexity to confidently navigate with human engineering, and (2) a guideline is needed for navigating this complexity. The above guideline is based on past experience, as viewed by the author, a non-specialist in the fields involved. The only more direct involvement of the author was in the development of procedure [70] for characterization of size distributions of conjugate anti-bacterial vaccines [70,71].

### 5.3. Support and Caution from Studies of Coronaviruses in Domestic Animals

Vaccines against coronaviruses have a relatively long history of use in domestic animals. These animals include dogs, cats, cattle, pigs and poultry. Successful vaccines display a profile similar to the profile in Table 1 and Table 2. That is to say, all vaccines licensed in North America are either LA or IW, as summarized in Table 1 of a 2020 review [72]. An RNA vaccine for porcine epidemic diarrhea virus is closest to breaking this pattern but has only been provisionally licensed by the US Department of Agriculture [72]. These results support the above guideline.

For one coronavirus infection of cats (but no coronavirus infections of the other domestic animals), problems with ADE have been found to be more extensive than apparent in the literature for humans. The coronavirus involved is feline infectious peritonitis virus [72]. Pre-existing antibodies, whether from a vaccine or not, cause virus uptake into macrophages that spread the infection [72,73,74]. A successful vaccine does not exist [72]. 

This problem, although rare with LA vaccines, does caution that further development of vaccine technology is likely to be necessary. What appears to be needed is a method for evolving the antigen so that it specifically binds to and elicits antibodies of the needed type (assuming that such antibodies are known) with minimal eliciting of antibodies of other types. This evolution appears most efficiently done with bacteriophage-associated antigens, given the relatively rapid reproduction of bacteriophages. Details for one possibility are described in reference 57. 

## 6. Implications: Bottom Line 

The statistical character of vaccine strategy is encapsulated by the following. Although one might execute a random walk through immune system complexity with a non-native vaccine, such as an unassembled protein vaccine, we should not be “betting the farm” (or the country or the world) on doing this rapidly. I note that RNA vaccines, DNA vaccines and even some vectored vaccines are designed to vaccinate with protein not assembled in capsids or other virus-related structures, although the unassembled protein is not the vaccine itself. Although perhaps forgotten, a similar thought was expressed by perhaps the foremost expert on vaccines. One example is the following quotation from 2002. “…. it seems a verifiable reality that simplification and reductionism may be inversely proportional to accomplishment because of the complexity and difficulty involved in their technical pursuit. Accomplishing a large immune response using less complex antigens, may be reaching a roadblock at the point of feasibility. This may be a warning for the future [75].” Post-reference 75, only papilloma vaccine appears, at first glance, to counter this skepticism. However, the nanoparticle, capsid-like character of the papilloma vaccine (above) makes this another apparent exception that supports the rule.

Furthermore, we have to face the fact that no RNA vaccine, DNA vaccine or unassembled, non-glycosylated peptide (introduced via a vector or not) anti-viral vaccine has ever been FDA approved in the United States. This conclusion is drawn from information provided by a website from the Center for Diseases Control in Atlanta. The approved anti-viral vaccines listed at this website have been mentioned above [76].

This negative result is not caused by lack of trying. Even by 2002, the efforts of the previous paragraph were, together, the dominant vaccine efforts [75]. Efforts to generate a mRNA rabies vaccine have been made for over nine years, motivated, in part, by the expense of LA rabies vaccines. However, sufficient reliability has not been achieved, in spite of the fact that an effective antigen (G glycoprotein) is known [6]. The delivery device used has a surprisingly high effect on the outcome. This might all be explained by digestion of the vaccine RNA by host RNases. In support, some improvement has been achieved by encapsulating the RNA in lipid nanoparticles [6]. DNA vaccines have the obvious limitation of potential chromosomal integration, with negative outcomes not detectable for years, possibly decades.

The bottom line is that, assuming current knowledge, success probability is projected to be maximized by vaccinating with an antigen that generates something as close to a real virus infection as safely possible.

## Figures and Tables

**Table 1:** Vaccines against ssRNA, membrane-enveloped viruses.

Virus	Year(s)	Vaccine Type(s)	References
Rabies	1885	LA, IW	[4,5,6]
Yellow Fever	1935–1937	LA	[4,7,8]
Japanese B Encephalitis	1944	IW, LA	[4,9,10]
Influenza	1945	LA, IW, IS, RS	[4,11,12,13]
Measles	1962	LA	[4,14,15]
Mumps	1966	LA	[4,16,17]
Rubella	1969–1979	LA	[4,18,19]

**Table 2:** Vaccines against other viruses. The single asterisk (*) refers to the only vaccine for animals. The double asterisk (**) refers to the unusual first vaccine for hepatitis B.

Virus	Year(s)	Vaccine Type(s)	References
Variola	1796	LA	[30,31]
Polio	1955	IW, LA	[4,32]
Adeno	1956–1957	IW	[4,33]
Marek’s Herpes *	1971	LA	[4,34]
Hepatitis B	1981	**, RS	[4,35,36]
Hepatitis A	1994	IW, LA	[4,37]
Varicella-Zoster	1995	LA	[4,38]
Rotavirus	2006	LA	[39,40]
Papilloma	2006	RS	[41,42]
